# A Scan for Positively Selected Genes in the Genomes of Humans and Chimpanzees

**DOI:** 10.1371/journal.pbio.0030170

**Published:** 2005-05-03

**Authors:** Rasmus Nielsen, Carlos Bustamante, Andrew G Clark, Stephen Glanowski, Timothy B Sackton, Melissa J Hubisz, Adi Fledel-Alon, David M Tanenbaum, Daniel Civello, Thomas J White, John J. Sninsky, Mark D Adams, Michele Cargill

**Affiliations:** **1**Biological Statistics and Computational Biology, Cornell UniversityIthaca, New YorkUnited States of America; **2**Center for Bioinformatics, University of CopenhagenDenmark; **3**Molecular Biology and Genetics, Cornell UniversityIthaca, New YorkUnited States of America; **4**Applied BiosystemsRockville, MarylandUnited States of America; **5**Celera GenomicsRockville, MarylandUnited States of America; **6**Celera DiagnosticsAlameda, CaliforniaUnited States of America; Sanger InstituteUnited Kingdom

## Abstract

Since the divergence of humans and chimpanzees about 5 million years ago, these species have undergone a remarkable evolution with drastic divergence in anatomy and cognitive abilities. At the molecular level, despite the small overall magnitude of DNA sequence divergence, we might expect such evolutionary changes to leave a noticeable signature throughout the genome. We here compare 13,731 annotated genes from humans to their chimpanzee orthologs to identify genes that show evidence of positive selection. Many of the genes that present a signature of positive selection tend to be involved in sensory perception or immune defenses. However, the group of genes that show the strongest evidence for positive selection also includes a surprising number of genes involved in tumor suppression and apoptosis, and of genes involved in spermatogenesis. We hypothesize that positive selection in some of these genes may be driven by genomic conflict due to apoptosis during spermatogenesis. Genes with maximal expression in the brain show little or no evidence for positive selection, while genes with maximal expression in the testis tend to be enriched with positively selected genes. Genes on the X chromosome also tend to show an elevated tendency for positive selection. We also present polymorphism data from 20 Caucasian Americans and 19 African Americans for the 50 annotated genes showing the strongest evidence for positive selection. The polymorphism analysis further supports the presence of positive selection in these genes by showing an excess of high-frequency derived nonsynonymous mutations.

## Abstract 2

Humans and chimps diverged about 5 million years ago. This study seeks to find the genes that have undergone positive selection during the evolution of both lineages since that time.

## Introduction

Genes, or regions of the genome, that have been affected by natural selection may show an excess of functionally important molecular changes, beyond what would be expected in the absence of selection. Genomic regions with such an excess of changes are said to have experienced positive selection, i.e., selection in favor of new genetic variants. The most common statistical technique for detecting positive selection takes advantage of the fact that mutations in coding regions of genes come in two classes: nonsynonymous mutations that change the resulting amino acid sequence of the protein and synonymous mutations, which do not change the encoded protein. An excess of nonsynonymous mutations over synonymous mutations, beyond what would be expected if the two types of mutations occur at the same rate, provides strong evidence for the past action of positive selection at the protein level. Using this logic, there have recently been numerous studies documenting positive selection in a variety of genes and organisms, including immune-response-related genes [1–3], viral genes [4–6], fertilization genes [7,8], and genes involved in sensory perception and olfaction in humans [9].

Clark et al. [10] compared 7,645 genes from humans to their orthologs from the chimpanzee and the mouse. For each gene, they tested if there was an excess of nonsynonymous substitutions on the evolutionary lineage leading to humans. They showed that there was an excess of putatively positively selected genes in several functional classes, including genes involved in sensory perception, olfaction, and amino acid catabolism. They also showed that human genes that have been targeted by positive selection are significantly more likely to harbor variation associated with known genetic diseases. We here report the results of an analysis of 20,361 human and chimpanzee genes (of which 6,630 later were eliminated in a very conservative quality control), which includes the 7,645 genes analyzed by Clark et al. [10]. While the objective of the study by Clark et al. [10] was to find genes that have experienced accelerated evolution on the human lineage, using the mouse as an outgroup, the aim of the current study is to find genes that have been targeted by positive selection at any point in time during the evolution of humans and chimpanzees, based on a larger set of genes. We use a likelihood ratio test to identify positive selection and do extensive simulations to find the appropriate critical values of the test. Positive selection is inferred if the ratio of nonsynonymous substitutions per nonsynonymous site to synonymous substitutions per synonymous site *(d_N_*/*d_S_)* is statistically significantly greater than one in a test of the neutral null hypothesis *d_N_*/*d_S_* = 1 [11,12]. The method used for detecting positive selection takes transition/transversion rate biases and unequal codon and amino acid frequencies into account. The test for positive selection applied in this study is a traditional test of *d_N_*/*d_S_* greater than one. It has more power than the test used in the Clark et al. study [10] if selection affects both the human and the chimpanzee lineages because it uses information from both lineages.

## Results

**Figure 1:**
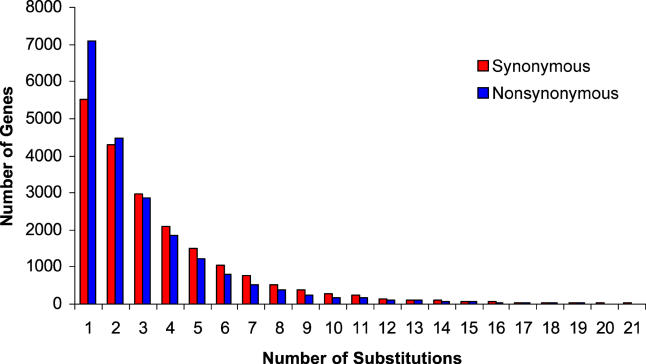
Distribution of Mutations The figure shows the number of synonymous and nonsynonymous nucleotide differences in 13,731 human–chimpanzee orthologous gene pairs.

Chimpanzee sequence was obtained by PCR using primers designed to flank exon sequence annotated in the human genome [10]. Our analysis begins with data from 20,361 coding regions, including 103,606 nucleotide differences and 403 indels among 17,687,331 aligned nucleotides. These numbers are significantly lower than the genome-wide averages [13,14], presumably due to selective constraints in the coding regions. The distributions of nonsynonymous and synonymous nucleotide differences among genes are shown in Figure 1. The average numbers of nonsynonymous and synonymous mutations per nucleotide site are 0.002578 and 0.003281, respectively. Eliminating reads without a hit to known genes in public databases (see Materials and Methods), there are 71,896 nucleotide differences in 13,731 genes. The remaining analysis is restricted to this set of genes. Among them, 5,574 were eliminated from the positive selection analysis because they had fewer than three mutations, and 797 were eliminated because the sequence was less than 50 bp long. Additionally, 45 genes were eliminated because they contained internal stop codons, presumably due to erroneous annotations or sequencing errors. Among the remaining 8,079 genes, 3,913 were also analyzed by Clark et al. [10].

The average level of sequence divergence was 0.60%, corresponding to a divergence level of 1.57% in silent sites. This figure matches well the level of divergence observed by Ebersberger et al. [14] for Chromosome 22 of 1.44% overall and 2.26% in CpG islands.

Seven hundred thirty-three of the 8,079 genes evolved with *d_N_*/*d_S_* greater than one, but only 35 had *p*-values less than 0.05, as determined by a likelihood ratio test of the null hypothesis of *d_N_*/*d_S_* = 1 against the alternative hypothesis of *d_N_*/*d_S_* greater than one. The number of significant genes at the 5% level, in this one-sided test, is lower than the nominal level because the vast majority of genes are conserved and evolve with *d_N_*/*d_S_* less than one. Nonetheless, after using Simes's improved Bonferroni procedure [15] we can, at the 5% significance level, reject the hypothesis that none of the genes are evolving with *d_N_*/*d_S_* greater than one. This also implies that a 5% false discovery rate set is nonempty. Even though the level of divergence between humans and chimpanzees is very low, there is statistically significant evidence for positive selection in the DNA sequences of these two species. Results for all genes are available in Dataset S1.

### Biological Processes Affected by Positive Selection

To identify functional groups of genes with an overrepresentation of putatively positively selected genes, we used the PANTHER [16,17] classification of biological processes and a Mann-Whitney *U* test (MWU) based on the *p*-values from the likelihood ratio test (Table 1). The classification based on the MWU identifies categories of genes with small *p*-values from the likelihood ratio test. It is important to notice that genes that evolve approximately neutrally will tend to have smaller *p*-values than genes evolving under strong functional constraints. The classification based on the MWUs, therefore, does not provide unambiguous evidence for positive selection, but it provides a key to which groups harbors the most candidates for positive selection.

**Table 1:**
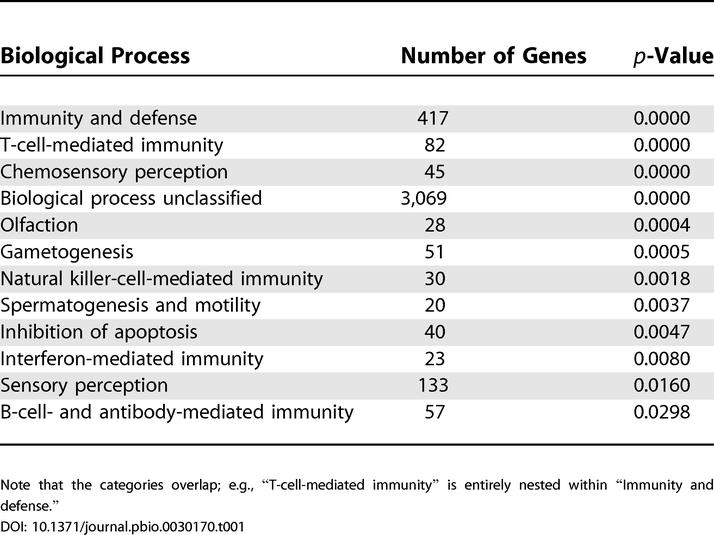
Biological Process Categories with an Excess of Putatively Positively Selected Genes (Nominal *p* less than 0.05; MWU) among a Total of 133 Biological Process Categories

Note that the categories overlap; e.g., “T-cell-mediated immunity” is entirely nested within “Immunity and defense.”

Immune-defense-related genes appear at the top of the list. It is not surprising that several of the genes experiencing most positive selection are involved in immune responses to viruses. Considering the speed at which many pathogens, such as viruses, evolve (e.g., [5]), a coevolutionary molecular arms race between pathogens and host cells might explain the presence of strong selection favoring new mutations in these genes. Other forces, including overdominant selection to diversify the spectrum of immune responses, may also cause positive selection in immune- and defense-related genes. Such explanations have previously been used to explain the presence of positive selection in the human major histocompatibility complex [18].

As in [10] we also identify genes involved in various forms of sensory perception, including olfaction and genes classified as “unknown biological function.” Many of the genes with unknown biological function show sequence similarity with known transcription factors (data not shown). Much of the selection on sensory genes is driven by the selection on olfactory receptors previously found by Gilad et al. [9].

In contrast to Clark et al. [10], we also find that genes involved in spermatogenesis appear to have an excess of positively selected genes. The genes involved in spermatogenesis showing the strongest evidence for positive selection include several KRAB-containing zinc finger proteins that serve as repressors of transcription and are believed to be involved in determining the differentiation of pluripotent stem cells [19].

### Expression Patterns and Positive Selection

We also categorized 3,464 of the 8,079 genes according to the tissue of expression in the Novartis Gene Expression Atlas [20]. Because of the relatively small number of tissue-selective genes in our dataset (204) and the large number of tissues analyzed (28), many tissues had fewer than 20 tissue-selective genes, providing little statistical power for further subdivision. Therefore, we examined instead whether the tissue of maximal expression for a gene was correlated with positive selection, since high expression levels and importance in tissue function are often, but not always, correlated. The set of genes that have their maximal expression in the testes is the only one showing an excess of positive selection, after a Bonferroni correction for multiple tests (Table 2).

**Table 2:**
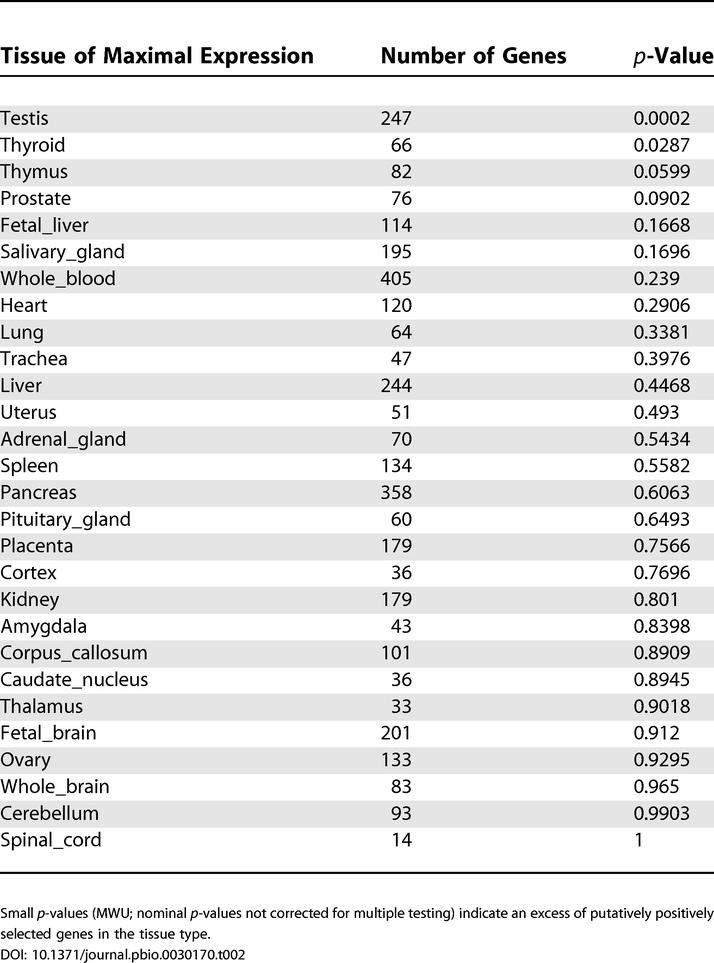
Test for an Excess of Putatively Positively Selected Genes by Tissue Type

Small *p*-values (MWU; nominal *p*-values not corrected for multiple testing) indicate an excess of putatively positively selected genes in the tissue type.

Genes with their maximal expression in the brain do not have an excess tendency toward positive selection. In fact, genes expressed in the brain seem to be among the most conserved genes with the least evidence for positive selection. MWUs, comparing genes with their maximal expression in the brain (83 genes) to all other genes, show that these genes tend to have significantly higher *p*-values of the likelihood ratio test for positive selection (*p* = 0.035), indicating high levels of selective constraint. Genes that are expressed in the brain at a level of twice the expression level found in blood show an even stronger tendency toward avoidance of positive selection (*p* = 0.0002). Although studies of gene expression in the brain tissue are complicated by low-abundance transcripts and heterogeneous specialized brain regions [21], the overall evidence points toward a deficiency of positively, or fast evolving, genes among those expressed in the brain. The causes for the cognitive differences may instead be sought in adaptive changes in just a few genes, in changes in gene expression [22], or in changes in copy number and/or organization of genes relating to cognitive function [23].

Dorus et al. [24] found that genes expressed in the nervous system showed a relative increase in the rate in primates relative to rodents when compared to housekeeping genes, but provided no direct evidence for positive selection on these genes. Nervous-system-specific genes appear to be so conserved that it is unlikely that direct evidence for positive selection will be discovered in this group of genes.

### Positive Selection in the X Chromosome

We also tested if any chromosomes show an excess of genes with evidence for positive selection. The only chromosome enriched in genes with small *p*-values from the likelihood ratio test for positive selection is the X chromosome (*p* = 0.0049; MWU). Several factors influence the contrast between the X and autosomes in tests of selection, including hemizygosity of the X in males, resulting in more effective selection against deleterious recessive and in favor of positive recessive mutations [25]. Male hemizygosity also results in mutations, with male-specific effects being more readily fixed by selection on the X [26]. This increased efficiency of selection for male-specific genes on the X may explain the excess of X-linked genes expressed in spermatogonia [27]. The observation that reproductive proteins generally evolve at a greater rate, coupled with the overrepresentation of male-specific genes on the X, could produce the excess positive selection seen on the X. However, after eliminating all genes with highest expression levels in the testis, or annotated as functioning in spermatogenesis, there is still an excess of putatively positively selected genes on the X chromosome (*p* = 0.0131; MWU). Thus, it appears that the elevated positive selection on the X is likely due to the general tendency of mutations to be recessive, regardless of their tendency to be male-limited in expression. Although other factors, such as an elevated male mutation rate [28], differences in the efficacy of genetic hitchhiking between autosomes and the X chromosome [29], and correlations between recombination rate and divergence [30], may cause differences in variability and substitution rate between autosomes and the X chromosome, none of these factors alone can explain the excess of positively selected genes on the X chromosome.

### Analysis of the 50 Genes Showing Strongest Evidence for Selection

We studied the 50 genes with the highest likelihood ratios in greater detail to further characterize the causes of positive selection and examine error rates (Table 3). To investigate the degree to which our results might be influenced by sequencing errors, we compared the data for these genes with the public data available for the same genes. In the regions with overlap between the public data and our data there were a total of 327 mutations in the public data and 306 mutations in our data. This demonstrates that there is not an excess of (potentially artifactual) mutations in our data in the genes that show evidence for positive selection. While most of the 50 genes also show strong evidence for positive selection in the public data, six of the genes do not. HC19953, HC2758, HC6579, HC7761, HC8067, and HC9844 do not have *d_N_*/*d_S_* ratios larger than one in the public data. In most cases, the difference is caused by the fact that our database and the public database contain different regions of the genes. Not all regions of a gene are expected to be targeted by positive selection, but this does not challenge the evidence for positive selection in the regions of the genes included in this analysis. In any case, using the public data would not change the qualitative conclusions of the analysis of the genes presented here.

**Table 3:**
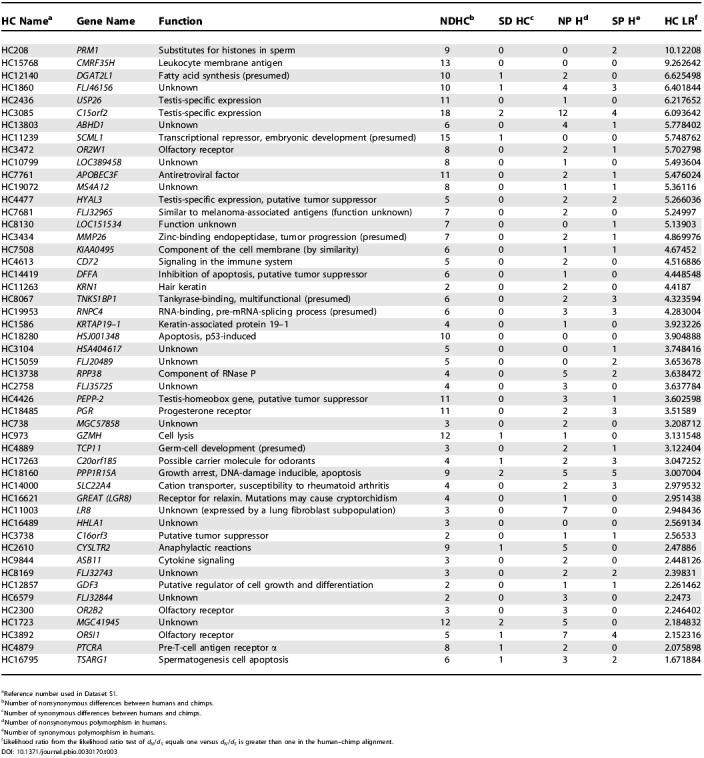
The Top 50 Genes Showing Evidence for Positive Selection

^a^Reference number used in Dataset S1.

^b^Number of nonsynonymous differences between humans and chimps.

^c^Number of synonymous differences between humans and chimps.

^d^Number of nonsynonymous polymorphism in humans.

^e^Number of synonymous polymorphism in humans.

^f^Likelihood ratio from the likelihood ratio test of *d_N_*/*d_S_* equals one versus *d_N_*/*d_S_* is greater than one in the human–chimp alignment.

### Immunity and Defense Genes Targeted by Positive Selection

The top 50 genes include many genes that we might a priori expect to be targets of positive selection, including four genes involved in olfaction *(OR2W1, OR5I1, OR2B2,* and *C20orf185)* and several genes involved in host–pathogen interactions, such as *CMRF35H, CD72* antigen, pre-T-cell antigen receptor α *(PTCRA), APOBEC3F,* and granzyme H *(GZMH)*. Only one of these genes was among the 50 most significant entries in the Clark et al. [10] model 2 analysis. *APOBEC3F* encodes an antiviral factor that has previously been demonstrated to be under positive selection by Sawyer et al. [3] who note that this gene has been associated with anti-HIV activity.

Presumably, most of these genes have been targeted by positive selection throughout the primate and mammalian phylogeny. The widespread evidence for positive selection in immune-related genes confirms the hypothesis that much positive selection in the human and mammalian genomes may be driven by a coevolutionary arms race between host immune system and pathogens.

### Spermatogenesis- and Apoptosis-Related Genes

The list also contains many testis- or sperm-specific genes including Protamine-1 *(PRM1),* which previously has been shown to be under positive selection [31], possibly due to sperm competition (but see [32] for an alternative explanation). Other sperm-specific genes on the list include *USP26, C15orf2, PEPP-2*, *TCP11, HYAL3,* and *TSARG1.* The inclusion of these genes in the list of the genes showing the strongest evidence for positive selection is consistent with the results, based on the PANTHER annotation and the Novartis expression data, of excess positive selection in sperm/testis-specific genes. The possible causes include sperm competition (e.g., [31]), sexual conflict (e.g., [7,8]), selection for reproductive isolation, pathogen-driven selection in the reproductive organs, and selection related to the occurrence of mutations causing segregation distortion.

We notice that at least one of these genes *(TSARG1)* is involved in apoptosis during spermatogenesis. Apoptosis of germ cells is conspicuous during normal spermatogenesis, eliminating up to 75% of the potential spermazoa [33–35], affecting cells both before and after the meiotic division [36]. It has been hypothesized that the main cause for the high rate of apoptosis during spermatogenesis is to maintain a proper cell-number ratio between maturing germ cells and Sertoli cells [35]. The natural process of elimination of germ cells by apoptosis creates a genomic conflict in which each individual germ cell will benefit from avoiding apoptosis, but apoptosis of a certain fraction of germ cells may be beneficial to the mature organism. New mutations occurring in cells during spermatogenesis, which reduces the probability of apoptosis, will be positively selected. This effect will be particularly strong for mutations in genes expressed after the meiotic division, potentially resulting in segregation distortion. A mutant with an even very small increase in the probability of escaping postmeiotic apoptosis will have a strong selective advantage. Compensatory mutations, reducing or eliminating the effect of the apoptosis avoidance mutation, may then later occur. These dynamics may lead to recurrent events of positive selection in genes affecting spermatogenesis apoptosis. The 40 genes in this study involved in inhibition of apoptosis show an excess of evidence for positive selection compared to other categories (*p* = 0.0047; see Table 2). Many of the genes showing most evidence for positive selection are known to be involved in either spermatogenesis, apoptosis, or both. For example, the apoptosis-related gene showing the strongest evidence for positive selection *(DFFA)* is an inhibitor of Fas-mediated apoptosis, which has been shown to be involved in apoptosis during spermatogenesis [36]. This may suggest that genomic conflict due to spermatogenesis apoptosis may be driving positive selection in many of the included genes.

### Cancer-Related Genes

While we expected to find genes involved in olfaction, spermatogenesis, and immune defense among the 50 annotated genes showing the strongest evidence for positive selection, we were surprised to find a very large proportion of cancer-related genes, especially genes involved in tumor suppression, apoptosis, and cell cycle control. These genes include four putative tumor suppressors: *HYAL3, DFFA, PEPP-2* (note that both *HYAL3* and *PEPP-2* also appear to be involved in spermatogenesis), and *C16orf3,* another gene associated with tumor progression *(MMP26)*, and a gene with unknown function but high similarity to melanoma-associated antigens *(FLJ32965).* In addition, there are several genes involved in apoptosis *(PPP1R15A, HSJ001348, TSARG1,* and *GZMH).* Given that many of the genes have very little functional information, it is surprising to find such a large proportion of genes that may be related to tumor development and control. The factors causing positive selection on these genes are unknown, but genes important in tumor development and suppression may be positively selected due to other functional effects of the genes, particularly in immunity and defense or in spermatogenesis. Several of the genes involved in tumor suppression or progression show testis-specific expression, and models of genomic conflict may explain the presence of positive selection in these genes. It should be noted that there is no pattern of human-specific selection in these genes. The high number of nonsynonymous mutations in these genes is approximately evenly distributed between the human and the chimpanzee lineage (results not shown).

### PAML Analysis

For each of the 50 genes, we searched public databases to find orthologous genes in other mammals. For 25 of the genes we were able to identify orthologs from mouse and rat, and for these 25 genes we estimated the *d_N_*/*d_S_* ratio of each lineage of the underlying phylogeny using PAML [37]. The *d_N_*/*d_S_* ratio was elevated (*p* < 0.05) in 5/25 cases in just the human lineage, in 5/25 cases in just the chimp lineage, in 8/25 cases in both lineages, and in 7/25 cases significant in neither lineage. These results show that the elevated *d_N_*/*d_S_* ratios are a consequence of positive selection in both the human and the chimpanzee lineage.

### Population Genetic Analysis

To further investigate the effect of selection on the 50 genes showing the strongest evidence for positive selection, 20 European-American and 19 African-American individuals were sequenced for these genes. Forty-six of the genes contained intraspecific polymorphism, and there were a total of 55 synonymous polymorphisms and 116 nonsynonymous polymorphisms, showing that the *d_N_*/*d_S_* ratio is also relatively high in the polymorphism data.

**Figure 2:**
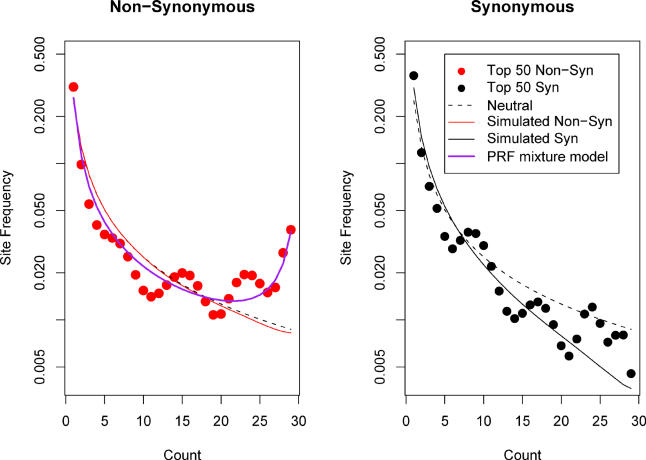
Frequency Spectra The figure shows the frequency spectra of nonsynonymous (red) and synonymous (black) mutations among the 50 genes showing the strongest evidence for positive selection in the interspecific comparison. Also shown is the expectation from the standard neutral model, expectations from the neutral model taking the protocol used to select the 50 genes into account (see text), and from the prediction of the selection model. On the *x*-axis is the number of derived allele in a sample of size 30 chromosomes (Count), and on the *y*-axis is the proportion of sites expected in the sample with a particular frequency.

The distribution of allele frequencies within these genes, as summarized by the allele frequency spectrum, provides additional support for positive selection. The frequency spectrum (Figure 2) of synonymous polymorphisms does not deviate from the pattern expected under a standard neutral model [38]. However, this does not necessarily provide evidence for the adequacy of the standard neutral model, but may rather be caused by a cancellation of effects due to population growth, population subdivision, and linkage to selected mutations, low power due to the small sample size, or by an ascertainment bias described below. Other data in humans have shown an excess of rare derived alleles in synonymous sites, presumably caused by population growth [39,40]. In contrast, we find that nonsynonymous single nucleotide polymorphisms (SNPs) show evidence for an excess of high-frequency-derived alleles in these genes (Figure 2). The excess of high-frequency-derived nonsynonymous mutation supports the notion that these genes have been targeted by positive selection. An important caveat is that an ascertainment bias has been introduced because interspecific and intraspecific variability has been confounded when selecting genes with high *d_N_*/*d_S_* ratios. To assess the impact of this ascertainment bias, we simulated 1,000 new neutral datasets, each dataset consisting of 13,731 genes with a similar distribution of *d_N_*/*d_S_* ratios, mutation rates, and sequence lengths, as observed in the real data, and with both interspecific and intraspecific variation. From these datasets we selected the 50 genes with the largest *d_N_*/*d_S_* ratios, as in the selection procedure applied to the real data. There is a clear effect of the ascertainment bias on synonymous sites, but there is essentially no effect on nonsynonymous sites (Figure 2). The main effect of the ascertainment bias is to eliminate genes with many high-frequency-derived synonymous mutations. This shows that the excess of high-frequency-derived nonsynonymous mutations is not a result of the ascertainment bias.

In addition to selection, certain demographic factors, such as population bottlenecks and population subdivision [41,42], and/or incorrectly inferred ancestral states may also enrich the sample with apparent high-frequency-derived mutations. Przeworski [42] has previously reported an excess of high-frequency-derived mutations in human data. To investigate this possibility we compared the frequency spectrum in our data to the frequency spectrum of the genes in the Seattle SNP database (SeattleSNPs; http://pga.gs.washington.edu [01/10/03]). These data also consist of a mixture of declared African Americans and European Americans and should, therefore, comprise a suitable sample for comparison. With 24 out of 116 and 37 out of 360 nonsynonymous mutations of frequency greater than 50% in our data and the Seattle data, respectively, there is a significant excess of high-frequency-derived mutations in our data compared to the Seattle data (*p* < 0.01, chi-square test). The Seattle data shows a slight deficiency of nonsynonymous-derived mutations with frequency greater than 50%, primarily due to an excess of very low-frequency-derived mutations. These results strongly suggest that the pattern we observe is caused by ongoing positive selection and not by demographic effects. There are a total of 25/78 and 22/92 polymorphisms of frequency greater than 50% within the Caucasian and African-American groups, respectively. Analyzing each population separately gives an even more extreme excess of high-frequency-derived polymorphism, especially in the Caucasian population.

There is a very high variance in the ratio of divergence to polymorphism in these genes (Hudson-Kreitman-Aguadé test; *p* less than 0.05). While the overall ratio of divergence to polymorphism is around two (2.06), a few genes stand out as having particularly high levels of polymorphism. For example, one of the olfactory receptors, *OR5I1,* has six substitutions and 11 polymorphisms. This raises the possibility that positive selection in the olfactory receptors may be a type of balancing selection. One possibility is heterozygote advantage driven by selection to increase the repertoire of olfactory receptors.

Another gene with a low divergence to polymorphism ratio is *RPP38* (four substitutions and seven polymorphisms), which is a subunit of RNase P. *RPP38* is necessary for normal processing of stable RNA in human cells, but it is also a target for antisera from systemic sclerosis patients. It is likely that the positive selection in this gene is caused by selection to avoid an autoimmune response. Such a hypothesis is plausible if the sequence pattern of RPP38 influences the likelihood of developing systemic sclerosis. This hypothesis can be tested using linkage or linkage disequilibrium studies.

Other genes show an apparent deficiency of polymorphisms. *SCML1* has 16 substitutions (of which 15 are nonsynonymous) and zero polymorphisms. Such a pattern is consistent with repeated selective sweeps driving divergence between species, while eliminating variation within species. *SCML1* is a repressor of expression of *Hox* genes and may play an important role in the control of embryonal development [43]. This gene may be a prime candidate for explaining developmental differences between humans and chimpanzees.

### Poisson Random Field (PRF) Analysis

To further investigate the distribution of selection coefficients among mutations in these genes, we applied a PRF model [44]. In PRF models, the distribution of sample allele frequencies can be expressed as a function of the scaled selection coefficient, *S,* (*S* = 2*Ns; N* = population size, *s* = selection coefficient) acting on a mutation. We assumed that there were three types of mutations: negatively selected mutations (of frequency *p*_–_), neutral mutations (of frequency *p*_0_), and positively selected mutations (of frequency *p_+_* = 1 – *p*_–_ – *p*_0_). We then estimated *p*_–_, *p*_0_, *p*_+_, and the scaled selection coefficients of the mutations in the two selected categories (*S*_–_ and *S*_+_) using maximum likelihood.

The maximum likelihood estimates of the parameters of the PRF model are *p*_–_ = 0.748, *p*_0_ = 0.172, *p*_+_ = 0.080, *S*_–_ = –34.96, and *S*_+_ = 267.11; i.e., the estimated proportion of negatively selected mutations is approximately 75%, and the proportion of positively selected mutations is approximately 8%. The proportion of positively selected mutations is so high because we have analyzed the 50 genes showing the strongest evidence for positive selection among a very large pool of candidate genes. Likelihood ratio tests show that a model with three selected classes fits the nonsynonymous data significantly better than a model with fewer selective classes (see Materials and Methods). We conclude that the allelic distribution in nonsynonymous sites is best described by a mixture of neutral, positively selected and negatively selected mutations. In this case, our best estimate of the proportion of mutations in these genes that are neutral is less than 18%. The predicted frequency spectrum under the estimated selection model is shown in Figure 2.

The results of this analysis should be interpreted with some caution because the effects of linkage have been ignored. The effects of linkage would be to underestimate the selection coefficient and, possibly, to overestimate the number of mutations that have been targeted by selection [45]. As previously discussed, these types of inferences are also sensitive to the demographic assumptions of a panmictic population of constant size [41,42] and to the assumptions regarding unambiguous inference of the ancestral state from the chimpanzee. For these reasons, the exact values of the parameter estimates should not be overinterpreted, but may help suggest the magnitude of the selective forces necessary to explain the data in isolation.

## Discussion

The statistical methods used for detecting selection have been the subject of debate over the past few years [46,47]. This debate has mainly focused on the validity of methods that model variation in the *d_N_*/*d_S_* ratio among sites. The current test does not model rate variation among sites and should, therefore, be uncontroversial. Unfortunately, this test may also have very low power.

**Figure 3:**
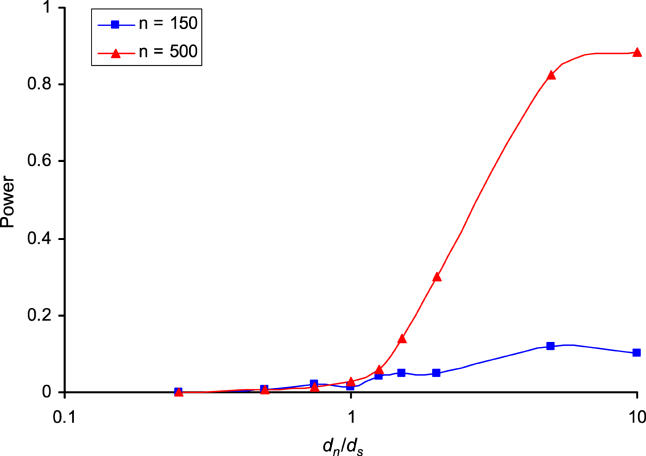
Power of the Likelihood Ratio Test for Positive Selection The power is shown as a function of the proportion of the *d_N_*/*d_S_* ratio, and for sequence lengths *(n)* of 150 and 500 codons. Power is defined as the proportion of tests that are significant at the 5% level. Simulation parameters, including codon frequencies, transition/transversion bias, and divergence times, are equal to the values estimated from the data. Notice the logarithmic *x*-axis.

To determine the power of the test, we conducted power simulations under parameter values estimated from the data (Figure 3). Notice first that the test does not result in excess significant results when *d_N_*/*d_S_* = 1, and results in very few falsely significant results when *d_N_*/*d_S_* is less than one. In fact, when *d_N_*/*d_S_* = 1 the power is lower than the nominal significance level because of the possibility of ties. However, the power increases steadily when *d_N_*/*d_S_* increases above one, and for a gene of length 500 codons, the test has more than 80% power when *d_N_*/*d_S_* = 5. For a functional gene, in which most sites are expected to be under functional constraints and evolve with *d_N_*/*d_S_* less than one, a significant value of the test is almost surely caused by positive selection. The fact that our data shows significant evidence for positive selection, when using this test with a correction for multiple tests, illustrates that positive selection can be detected from human–chimpanzee comparative data despite the very low levels of divergence.

In the previous study by Clark et al. [10], an outgroup (mouse) was used to make inferences regarding human-specific processes. We have here analyzed a larger dataset but cannot, in general, distinguish between selection that is particular to the human evolutionary lineage and positive selection that tends to occur in both chimps and humans. While Clark et al. [10] found strongest evidence for positive selection in genes related to olfaction and sensory perception, we find the strongest evidence for positive selection in genes related to immunity and defense. The reason is probably that genes related to immunity and defense are targets for positive selection throughout the mammalian phylogeny, which the test used by Clark et al. [10] would not detect, whereas much of the selection on sensory perception and olfaction is likely to be specific to the distinct niches of humans and chimpanzees. Similar arguments may also explain why we obtain strong evidence for positive selection on genes related to spermatogenesis and inhibition of apoptosis, while Clark et al. [10] did not find any evidence for human-specific selection on genes related to spermatogenesis and apoptosis.

In this paper we analyzed population genetic data from the 50 genes showing most evidence for positive selection. An excess of high-frequency-derived nonsynonymous mutations in these data supports the conclusions that these genes are targeted by positive selection. Although some demographic models also may cause an excess of high-frequency-derived mutations [41,42], the excess observed in our data is beyond the level observed in other comparable human data.

The use of the population genetic data may also help suggest the mode of positive selection acting on the gene. For example, a developmental gene *(SCML1)* had 16 fixed substitutions and zero polymorphisms, suggesting repeated selective fixations, whereas and olfactory receptor had six substitutions and 11 polymorphisms consistent with the action of balancing selection. The combined use of comparative and population genetic data may help, not only to identify positive selection, but also to help narrow down possible models of positive selection. With the increased availability of both comparative genomic data and SNP data, we expect to see many future studies that take advantage of the availability of both types of data.

The discovery that many genes involved in spermatogenesis, apoptosis, and tumor suppression are positively selected may prompt further investigations into models of genomic conflict and other models predicting positive selection in these genes. In general, mutations that increase the expected number of functional sperm cells produced by a specific germ-line cell, such as mutations increasing the rate of cell division or decreasing the probability of apoptosis, will be favored. Such mutations will not necessarily increase the fitness of the mature organism, leading to a genomic conflict, in which selfish mutations causing avoidance of apoptosis are being counteracted by compensatory mutations in other loci. Many of the genes with evidence for positive selection encountered in this study play functional roles in cell cycle regulation, tumor suppression, apoptosis, or spermatogenesis. We suggest that a genomic conflict relating to the process of spermatogenesis may be responsible for much of the positive selection observed in this study. Because many of these genes are involved in inhibition of apoptosis, this may also explain the apparent excess of cancer-related genes targeted by positive selection. This raises the interesting prospect that the high prevalence of cancer in humans and other organisms may be related to selection for apoptosis avoidance in the germ line. Mutations that in general increase apoptosis avoidance will be selected in the germ line, but such mutations may at the same time increase the probability of cancer in somatic tissue. The relative high prevalence of cancer will, according to this hypothesis, be related to an evolutionary conflict between the selfish interests of a germ cell and selection at the level of mature organisms to decrease the cancer rate. We note that the fact that the same pathways (e.g., Fas-mediated apoptosis) are involved in the control of cancer and in apoptosis during spermatogenesis supports this hypothesis.

At present we cannot exclude an alternative hypothesis, such as pathogen-driven positive selection or sperm competition. Future functional and evolutionary studies of the genes suggested to be under positive selection by this study may help determine which of these alternative evolutionary models are most plausible.

## Materials and Methods

### 

#### DNA sequencing and alignment.

Sequences of chimpanzee genes were obtained by PCR amplification of individual exons from a single western chimpanzee male. PCR products were directly sequenced on automated sequencers at Celera Genomics. Details of primer construction, DNA sequencing, and alignment were described in Clark et al. [10] and references therein. Chimpanzee sequences were obtained for both strands from PCR products, filtered to remove base calls with Phred scores less than 30. Genes that did not have a hit in the curated accessions (NM_ or NR_ series) in the REFSEQ 3.0 database (http://www.ncbi.nlm.nih.gov/RefSeq/) and for which the best hit did not map to the same genomic location in NCBI 34 build of the human genome, were omitted from the functional analysis, to minimize the chance of including nonfunctional genes/regions. This was done using BLAT v. 27 (http://www.genomeblat.com/genomeblat/index.asp). Regions of the alignment that were not in the REFSEQ database were eliminated from the analysis. However, we provide the full dataset (Dataset S2) for future exploration. Indels were identified after alignment as all nonterminal gaps that could not be attributed to low base-calling scores. All pairwise alignments are available in Phylip and FASTA format in Dataset S2.

Human polymorphisms were detected automatically from assembled sequencing traces using PolyPhred 4.0 [48] and RuleGen, a decision-tree-based method (S. Glanowski, unpublished data). Manual calls were employed if a potential SNP was not flagged by both programs. Validation of the automated pipeline using a set of several hundred manually called SNPs showed a sensitivity of 85% for all SNPs and up to 100% for SNPs with more than three minor alleles observed. Independent verification of several hundred SNPs using TaqMan assays indicated that validation rates of 95% for common SNPs and 90% for SNPs with only one minor allele were observed.

#### Likelihood ratio tests

For each human–chimpanzee orthologous gene pair, a likelihood ratio test of the hypothesis of an equal *d_N_*/*d_S_* ratio was performed using a codon-based likelihood model (see [12] for such tests). The test was performed as a one-sided test of the hypothesis H_0_: *d_N_*/*d_S_* = 1 versus the alternative of H_A_: *d_N_*/*d_S_* greater than one. To reduce the computational burden, the transition/transversion rate ratio was first estimated for genes in high-GC-content regions and low-GC-content regions separately. This parameter was then considered fixed for the remainder of the analysis. Because many of the sequence pairs showed very little divergence, the usual asymptotic assumptions of a chi-square distribution of the likelihood-ratio test statistic would not have been appropriate. Instead, simulations were performed to determine the appropriate distribution of the test statistic. The simulations were performed under the empirical distribution of the divergence time and other parameter estimates assuming *d_N_*/*d_S_* = 1. The distribution of the test statistic, conditional on the observed number of nucleotide differences between the sequences (for each gene), was then determined. One of the advantages of using the conditional distribution is that the distribution becomes more robust to violations of the assumptions regarding the nuisance parameters, particularly the divergence times, and this will allow us to exclude genes with very little variability while maintaining the right size of the test. Genes with fewer than three nucleotide differences, or with fewer than 50 aligned codons, were excluded from the analysis.

#### Functional analysis.

The functional annotation was performed as in [10], using the PANTHER database [16,17]. Throughout, excesses of positively selected genes in a category were tested, using an MWU comparing the distribution of *p*-values obtained from the likelihood ratio tests in genes included in the category to the distribution of such values in genes not included in the category. Genes with fewer than three nucleotide differences between humans and chimpanzees were excluded from the identification of categories with an excess of putatively positively selected genes. The MWU does not in itself demonstrate that the evolution of a particular category of genes is affected by positive selection, but it shows that the category contains more evidence for positive selection than other genes in the study. Because genes of short sequence length are less likely to show strong evidence for positive selection, but are more likely to show spurious evidence for positive selection, the MWU (or any other categorization) may be affected by different sequence lengths in different categories. The reason for using an MWU, instead of reporting overall *p*-values for a category after correction for multiple testing, is that such an approach would be strongly influenced by just one or a few genes. However, correction for multiple testing reveals significant positive selection in several categories, including the immune and defense and the spermatogenesis category.

#### Expression data.

Expression data from normal human tissues were obtained from the Novartis Gene Expression Atlas ([20]; http://wombat.gnf.org/index.html; 6,741 gene symbols could be matched unambiguously to the human–chimp alignments. All negative expression and values less than 20 were coded as 20. Tissue selectivity was determined by averaging probe expression values across samples and replicate tissues. In total, 61 samples were collapsed into 28 tissues. A probe was classified as tissue selective if it was expressed in only one tissue at a value of 200 or higher, and all other tissues were less than 100. Probes were then collapsed into genes. A gene was classified as tissue selective if at least one of its probes showed specificity. The tissue of maximal expression was determined by identifying the probe and sample (*n* = 61) with the highest expression value that was greater than 20 (85% of the genes had values greater than 200). Probes were then collapsed into genes. Tissue expression was determined by averaging the sample replicates (*n* = 28). A gene was considered expressed in a tissue if its expression value was greater than 200.

#### PAML analysis.

To obtain orthologous sequences for the 50 annotated genes with the highest likelihood ratios, we downloaded “Unique Best Reciprocal Hits” between human and mouse and human and rat from the Ensembl Web site (http://www.ensembl.org/). Sets of human, mouse, rat, and chimpanzee sequences were translated and aligned using ClustalW [49]. Codon alignments were generated using the ClustalW alignments as a guide, then manually checked. Partial sequences covering less than 80% of the human sequence were eliminated, and ambiguously aligned regions were masked before analysis. The underlying phylogeny was assumed to be ([chimpanzee, human], [mouse, rat]) for all genes.

The lineage-specific analysis was done in PAML [37] by allowing two values of the *d_N_*/*d_S_* ratio along the lineages of the phylogeny, one for the human lineage and one for all other lineages. To test if the *d_N_*/*d_S_* ratio was different on the human lineage, we then compared the maximum likelihood value in this model to the maximum likelihood value obtained, assuming the *d_N_*/*d_S_* ratio was constant among lineages. If two times the log likelihood ratio was larger than 3.84, we rejected the model of constant *d_N_*/*d_S_* ratio at the 5% significance level. This analysis was then repeated using the chimpanzee lineage as the focal lineage instead of the human lineage.

#### Calculating the frequency spectrum.

Because of missing data for many polymorphisms, the frequency spectrum in a sample of size 30 is reported. The frequency spectrum was calculated in a sample of size 30 as



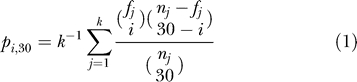



where *p_i,_*_30_ is the frequency of SNPs with derived alleles that exist in *i* copies in a sample of size 30, *n_j_* is the chromosomal sample size of the *j*th SNP, *f_j_* is the frequency of the derived allele for the *j*th SNP, *k* is the number of SNPs, and (^*i*^_*j*_) = 0 = 0 --> if *i* is less than *j*. The polarity of the mutation was determined using the chimpanzee sequence as outgroup.

#### Analysis of ascertainment bias.

To assess the impact of the ascertainment scheme in the tests that contrast human polymorphism data to the human–chimp divergence, new datasets were simulated, using standard neutral coalescence simulations (e.g., [38]). Each simulated dataset generated one chimp sequence and 78 human sequences for each of the 13,731 genes. For each simulated gene, one human sequence was randomly chosen and compared to the chimp sequence using a chi-square statistic for the goodness-of-fit test of *d_N_*/*d_S_* = 1. The 50 genes with largest chi-square statistic among genes with *d_N_*/*d_S_* greater than one were selected for population genetic analysis. This scheme was repeated 1,000 times to investigate the effect of the ascertainment protocol of the 50 genes. The parameters of the simulations were estimated from the data, using the observed distribution of sequence lengths, and synonymous-site mutation rate and humans–chimp divergence time estimated from the concatenated data. The distribution of *d_N_*/*d_S_* ratios among genes was estimated assuming the *d_N_*/*d_S_* ratios follow a γ distribution among genes, keeping the synonymous rate constant among them.

#### Power analysis.

To analyze the power of the test for positive selection, we simulated pairs of sequences and performed likelihood ratio tests of H_0_: *d_N_*/*d_S_* equals one versus *d_N_*/*d_S_* is greater than one for each sequence pair. The simulations were done using the average value of synonymous sequence divergence observed in the data, while nonsynonymous divergence was varied. For more details regarding such simulations, see, e.g. [50].

#### PRF analysis.

Assume nonlethal mutations enter a population of constant size 2*N* according to a Poisson process and are assigned to one of three categories: neutral (*S* = 0), positively selected with selection coefficient *S*_+_, and negatively selected with selection coefficient *S*_–_, according to probabilities *p*_0_, *p*_+_, and *p*_–_ (where *p*_0_ + *p*_+_ + *p*_–_ = 1). Furthermore, assume mutations evolve independently. It follows from standard population genetic theory, the total law of probability, and the rules of conditional probability that the probability of an SNP being found at frequency *i* out of *n* chromosomes under this scheme [44] is



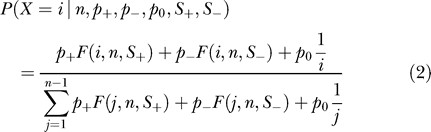



where *F*(*i,n,S*) --> is given by



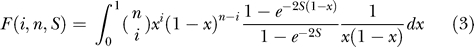



The likelihood of observing counts *x*_1_, *x*_2_, . . ., *x_S_* where *S* is the total number of segregating sites out of *n*_1_, *n*_2_, …, *n_S_* chromosomes is, thus,



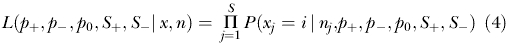



The maximum likelihood value and the maximum likelihood parameter estimates can then be obtained by numerically maximizing this function with respect to the parameters. Likelihood ratio tests can be constructed by constraining certain of the parameters to take on particular values. For example, setting *p*_0_ = 1 defines a model with no selected mutations. Likewise, setting *p*_0_ + *p*_–_ = 1 defines a model that allows negative selection, but no positive selection.

This analysis assumes that mutations are independent. Because of linkage and the possibility of epistasis, the independence assumption may not be met by the data. However, a full analysis taking the correlation among SNPs into account is not computationally feasible. Fortunately, the average correlation is low between SNPs because they have been sampled among 50 genes distributed throughout the genome. The effect of the correlation among SNPs on this analysis should, therefore, be minimal.

The maximum log likelihood value for the full model is –234.19. However, the maximum log likelihood values for models assuming only neutral mutations, or a single class of selected mutations, are –243.82 and –240.88, respectively. Under the independence assumption, both of these simpler models can be rejected against the model with three classes of mutations, using a likelihood ratio test (*p* = 0.0006 and *p* = 0.004).

## Supporting Information

Dataset S1 Results File (3.1 MB XLS).

Dataset S2 Alignment File (9.8 MB ZIP).

### Accession Numbers

The sequence analyzed in this study has been submitted to GenBank (http://www.ncbi.nlm.nih.gov/Genbank/).

## Abbreviations

*(d_N_*/*d_S_)*: ratio of nonsynonymous substitutions per nonsynonymous site to synonymous substitutions per synonymous site
MWU: Mann-Whitney *U* test
PRF: Poisson random field
SNP: single nucleotide polymorphism
